# Childhood asthma diagnoses declined during the COVID-19 pandemic in the United States

**DOI:** 10.1186/s12931-023-02377-7

**Published:** 2023-03-10

**Authors:** Daniel B. Horton, Amanda L. Neikirk, Yiling Yang, Cecilia Huang, Reynold A. Panettieri, Stephen Crystal, Brian L. Strom, Lauren E. Parlett

**Affiliations:** 1 Department of Pediatrics, Rutgers Robert Wood Johnson Medical School, Rutgers University, New Brunswick, NJ USA; 2 Department of Biostatistics and Epidemiology, Rutgers School of Public Health, Rutgers University, Piscataway, NJ USA; 3 Center for Pharmacoepidemiology and Treatment Science, Institute for Health, Health Care Policy and Aging Research, Rutgers University, 112 Paterson Street, New Brunswick, NJ 08901 USA; 4 HealthCore Inc., Wilmington, DE USA; 5 Rutgers Institute for Translational Medicine and Science, New Brunswick, NJ USA; 6 Department of Medicine, Rutgers Robert Wood Johnson Medical School, New Brunswick, NJ USA; 7 Rutgers Center for Health Services Research, Institute for Health, Health Care Policy and Aging Research, Rutgers University, New Brunswick, NJ USA; 8 School of Social Work, Rutgers University, New Brunswick, NJ USA; 9 Rutgers Biomedical and Health Sciences, Newark, NJ USA

**Keywords:** Asthma/epidemiology, Child, Adolescent, Pandemics, Database

## Abstract

**Background:**

Prior studies have documented declines in pediatric asthma exacerbations and asthma-related health care utilization during the COVID-19 pandemic, but less is known about the incidence of asthma during the pandemic.

**Methods:**

We conducted a retrospective cohort study of children under age 18 without a prior diagnosis of asthma within a large US commercial claims database. Incident asthma was defined using a combination of diagnosis codes, location of services, and medication dispensing. Crude quarterly rates of asthma diagnosis per 1000 children were calculated, and the incidence rate ratio and 95% confidence interval were estimated for newly diagnosed asthma during versus before the pandemic using negative binomial regression, adjusted for age, sex, region, and season.

**Results:**

Compared with 3 years prior to the pandemic, crude incident diagnosis rates of asthma decreased by 52% across the first four quarters of the US pandemic. The covariate-adjusted pandemic-associated incidence rate ratio was 0.47 (95% confidence interval 0.43, 0.51).

**Conclusions:**

New diagnoses of childhood asthma in the US declined by half during the first year of the pandemic. These findings raise important questions whether pandemic-related changes in infectious or other triggers truly altered the incidence of childhood asthma beyond the well-described disruptions in healthcare access.

## Background

The COVID-19 pandemic has caused profound interruptions in healthcare access and delivery [1, 2]. Prior studies have documented declines in pediatric asthma exacerbations and asthma-related emergency department usage and hospitalization [2, 3]. Only one previous paper studied incident asthma diagnoses in children [4]. However, this study from Japan included data only from selected facilities with complete electronic medical record data, identified patients based on a single asthma diagnosis, and did not evaluate incidence rates of diagnosis based on a known denominator.

Given the unanswered questions about asthma diagnoses in children during the COVID-19 pandemic, we sought to determine whether there were pandemic-related declines in the incidence of new asthma diagnoses in children in the United States.

## Methods

### Aim, design and setting

To examine whether the incidence of new asthma diagnoses in children in the United States changed during the early stages of the COVID-19 pandemic, we performed a retrospective cohort study. We conducted this study using the HealthCore Integrated Research Database (March 2016–February 2021), a large US commercial claims database.

### Study population

We identified members under 18 years old with ≥ 12 months of baseline continuous enrollment without a prior diagnosis of asthma. Incident asthma was defined using a combination of International Classification of Diseases, Version 10, Clinical Modification (ICD-10-CM) diagnosis codes (J45) and location and timing of medical services (≥ 1 inpatient hospitalization, ≥ 2 outpatient visits ≥ 8 weeks apart, or ≥ 1 outpatient visits plus dispensing of disease-specific medications within 1 month). This algorithm was adapted from previously validated algorithms with positive predictive values ≥ 90% [5].

### Comparison

We compared the crude and adjusted rates of new asthma diagnoses during the first year of the US COVID-19 pandemic with rates of new asthma diagnoses during the prior 3 years.

### Statistical analysis

Crude rates of asthma diagnosis per 1,000 children per quarter with 95% confidence intervals (CIs) were calculated from 2017 to 2021. Each quarter was 90 days in length and anchored on March 1, 2020, considered the start of the first quarter of the pandemic. Incidence rates during the first year of the pandemic (March 2020–February 2021) versus 3 years before the pandemic (March 2017–February 2020) were modeled using multivariable negative binomial regression with the SAS command, PROC GENMOD. Regression models were adjusted for the following covariates: age group (categorized as 1–5, 6–11, or 12–17), sex (male or female), region (Midwest, Northeast, South, West, or Missing), and quarter (quarters 1–4, to account for seasonality). Models used offsets of the log of children per quarter. Regression coefficients were exponentiated to obtain incidence rate ratios (IRRs) with Wald 95% CI. Analyses were conducted using SAS version 9.4.

## Results

**Fig. 1:**
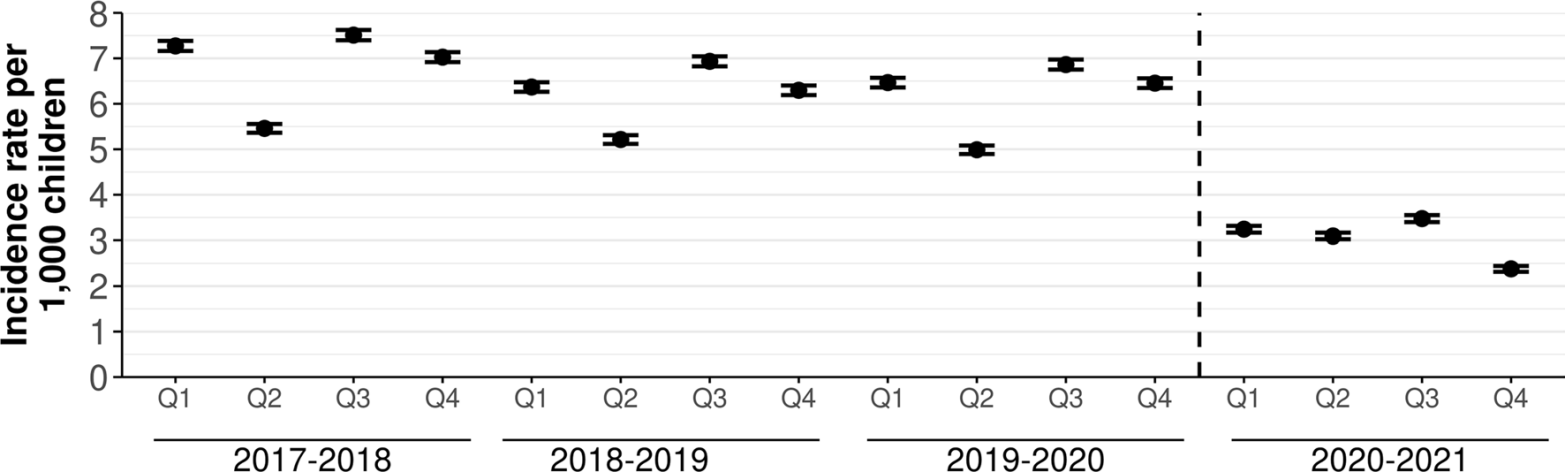
Quarterly incident diagnosis rates of childhood asthma, March 2017–February 2021. Crude quarterly diagnosis rates of asthma per 1000 children (circles) and 95% confidence intervals (bars). Dashed line indicates start of the US COVID-19 pandemic in March 2020

Compared with the 12-quarter pre-pandemic period, crude incident diagnosis rates of asthma decreased by 52% across the first four quarters of the US pandemic (pandemic 3.05 per 1000 children [95% CI 2.30–3.80] vs. pre-pandemic 6.40 [95% CI 5.89–6.92]) (Fig. 1).

The covariate-adjusted pandemic-associated IRR aligned with the observed crude rates: 0.47 (95% CI 0.43, 0.51) (Table 1).

**Table 1:** Incident diagnosis rate ratios of childhood asthma, March 2017–February 2021

Characteristic	Level	IRR^a^	95% CI
Age Group (reference = 1–5 yo)	6–11 yo	1.21	1.10, 1.33
	12–17 yo	1.41	1.28, 1.56
Sex (reference = Female)	Male	1.26	1.16, 1.36
Region (reference = West)	Midwest	0.84	0.74, 0.95
	Northeast	1.05	0.93, 1.19
	South	1.05	0.93, 1.19
	Missing	1.47	1.30, 1.67
Quarter (reference = Q1)^b^	Q2	0.80	0.72, 0.89
	Q3	1.10	0.98, 1.23
	Q4	0.86	0.77, 0.96
Pandemic (reference = pre-pandemic)^c^	Pandemic	0.47	0.43, 0.51

*CI* confidence interval, *IRR* incidence rate ratio, *Q* quarter, *yo* years old

^a^IRR modeled by multivariable negative binomial regression

^b^Average effects of Q2, Q3, and Q4 vs. Q1 across all years 2017–2021

^c^Pre-pandemic = March 2017–February 2020; pandemic = March 2020–February 2021

## Discussion

In a large, geographically diverse pediatric US population, new asthma diagnoses declined by half among children over the first 12 months of the COVID-19 pandemic. These findings underscore a dramatic change in the incidence of asthma in the US during the early phases of the pandemic, complementing other literature on pandemic-related improvements in asthma control.

Prior studies on children with pre-existing asthma documented large reductions in asthma exacerbations and asthma-related use of emergency services during the pandemic [2, 3, 6]. While the pandemic led to notable disruptions in healthcare access and delivery[1, 2], the apparent improvements in asthma control may have also occurred, in part, to lower exposures to circulating respiratory viruses, such as rhinovirus [7], and other environmental triggers[8].

In contrast to the many studies that have focused on pediatric patients and populations previously diagnosed with asthma, much less has been published on the incidence of asthma during the pandemic. To our knowledge, only one prior study has investigated new cases of asthma: a Japanese study showing declines in the number of pediatric asthma diagnoses across multiple facilities in the first 15 months of the pandemic [4]. These authors also showed a correlation between declines in asthma incidence and declines in numbers of documented cases of respiratory syncytial virus and rhinovirus. In contrast to that study, our study was population-based, performed in a general US population, estimated actual changes in incidence, and used a robust outcome definition.

Viruses such as rhinovirus are well-described triggers of childhood wheezing and subsequent diagnoses of asthma in children [9]. Notably, young children with allergic sensitization are more susceptible to rhinovirus-associated wheezing [10]. Rhinovirus infection may also predispose some children to develop asthma, particularly in the presence of certain commensal respiratory microbiota, such as *Moraxella*, *Haemophilus*, and *Streptococcus*.[11, 12] We posit that physical distancing and wearing of masks in the early stages of the pandemic limited exposure to and inhalation of asthma-inducing respiratory viruses. In various surveillance studies, a substantial reduction in the circulation of rhinovirus and other non-SARS-CoV-2 respiratory viruses was observed early in the pandemic, coinciding with protective measures such as lockdown and school closures [13, 14]. Given the role of pollutants in the development of childhood asthma [15], pandemic-associated declines in air pollution may also have contributed to the observed decrease in asthma incidence [16, 17].

A limitation of our study includes possible misclassification of either asthma diagnosis or newly diagnosed asthma based on our diagnostic algorithm. However, it was based on previously validated and highly accurate algorithms [5]. Additionally, our study did not include more recent data on asthma incidence in the US.

## Conclusion

In conclusion, new diagnoses of childhood asthma in the US declined by half during the first year of the pandemic. These findings raise important questions whether pandemic-related changes in infectious or other triggers truly altered the incidence of childhood asthma beyond the well-described disruptions in healthcare access. Research on disease etiology and more recent diagnostic trends and clinical outcomes of asthma will help answer these questions.

## Data Availability

Commercial claims data used for this study are not publicly available due to restrictions imposed by existing or anticipated agreements.

## Abbreviations

CI: Confidence interval
ICD-10-CM: International Classification of Diseases, Version 10, Clinical Modification
IRR: Incidence rate ratio

## References

[1] Moynihan R, Sanders S, Michaleff ZA, Scott AM, Clark J, To EJ, Jones M, Kitchener E, Fox M, Johansson M (2021). Impact of COVID-19 pandemic on utilisation of healthcare services: a systematic review. BMJ Open.

[2] DeLaroche AM, Rodean J, Aronson PL, Fleegler EW, Florin TA, Goyal M, Hirsch AW, Jain S, Kornblith AE, Sills MR, et al. Pediatric emergency department visits at US children’s hospitals during the COVID-19 pandemic. Pediatrics. 2021; 147. 10.1542/peds.2020-039628 33361360

[3] Hurst JH, Zhao C, Fitzpatrick NS, Goldstein BA, Lang JE (2021). Reduced pediatric urgent asthma utilization and exacerbations during the COVID-19 pandemic. Pediatr Pulmonol.

[4] Matsumoto N, Kadowaki T, Takanaga S, Ikeda M, Yorifuji T (2022). Impact of COVID-19 pandemic-associated reduction in respiratory viral infections on childhood asthma onset in Japan. J Allergy Clin Immunol Pract.

[5] Yousif A, Dault R, Courteau M, Blais L, Cloutier AM, Lacasse A, Vanasse A (2022). The validity of diagnostic algorithms to identify asthma patients in healthcare administrative databases: a systematic literature review. J Asthma.

[6] Yang Z, Wang X, Wan XG, Wang ML, Qiu ZH, Chen JL, Shi MH, Zhang SY, Xia YL (2022). Pediatric asthma control during the COVID-19 pandemic: a systematic review and meta-analysis. Pediatr Pulmonol.

[7] Taquechel K, Diwadkar AR, Sayed S, Dudley JW, Grundmeier RW, Kenyon CC, Henrickson SE, Himes BE, Hill DA (2020). Pediatric asthma health care utilization, viral testing, and air pollution changes during the COVID-19 pandemic. J Allergy Clin Immunol Pract.

[8] Eguiluz-Gracia I, Mathioudakis AG, Bartel S, Vijverberg SJH, Fuertes E, Comberiati P, Cai YS, Tomazic PV, Diamant Z, Vestbo J (2020). The need for clean air: the way air pollution and climate change affect allergic rhinitis and asthma. Allergy.

[9] Jackson DJ, Gern JE (2022). Rhinovirus infections and their roles in asthma: etiology and exacerbations. J Allergy Clin Immunol Pract.

[10] Jartti T, Kuusipalo H, Vuorinen T, Soderlund-Venermo M, Allander T, Waris M, Hartiala J, Ruuskanen O (2010). Allergic sensitization is associated with rhinovirus-, but not other virus-, induced wheezing in children. Pediatr Allergy Immunol.

[11] Teo SM, Tang HHF, Mok D, Judd LM, Watts SC, Pham K, Holt BJ, Kusel M, Serralha M, Troy N (2018). Airway microbiota dynamics uncover a critical window for interplay of pathogenic bacteria and allergy in childhood respiratory disease. Cell Host Microbe.

[12] Tang HHF, Lang A, Teo SM, Judd LM, Gangnon R, Evans MD, Lee KE, Vrtis R, Holt PG, Lemanske RF (2021). Developmental patterns in the nasopharyngeal microbiome during infancy are associated with asthma risk. J Allergy Clin Immunol.

[13] Haapanen M, Renko M, Artama M, Kuitunen I (2021). The impact of the lockdown and the re-opening of schools and day cares on the epidemiology of SARS-CoV-2 and other respiratory infections in children—a nationwide register study in Finland. EClinicalMedicine.

[14] Partridge E, McCleery E, Cheema R, Nakra N, Lakshminrusimha S, Tancredi DJ, Blumberg DA (2021). Evaluation of seasonal respiratory virus activity before and after the statewide COVID-19 shelter-in-place order in Northern California. JAMA Netw Open.

[15] Khreis H, Kelly C, Tate J, Parslow R, Lucas K, Nieuwenhuijsen M (2017). Exposure to traffic-related air pollution and risk of development of childhood asthma: a systematic review and meta-analysis. Environ Int.

[16] Berman JD, Ebisu K (2020). Changes in U.S. air pollution during the COVID-19 pandemic. Sci Total Environ.

[17] Chen LA, Chien LC, Li Y, Lin G (2020). Nonuniform impacts of COVID-19 lockdown on air quality over the United States. Sci Total Environ.

